# Comparative Assessment of Cytokine Pattern in Early and Late Onset of Neonatal Sepsis

**DOI:** 10.1155/2017/8601063

**Published:** 2017-03-05

**Authors:** Kh. S. Khaertynov, S. V. Boichuk, S. F. Khaiboullina, V. A. Anokhin, A. A. Andreeva, V. C. Lombardi, M. A. Satrutdinov, E. A. Agafonova, A. A. Rizvanov

**Affiliations:** ^1^Kazan State Medical University, Kazan, Russia; ^2^Kazan Federal University, Kazan, Russia; ^3^Nevada Center for Biomedical Research, Reno, NV, USA; ^4^Republic Children's Clinical Hospital, Kazan, Russia

## Abstract

Neonatal sepsis is a significant health issue associated with high mortality. Immune responses associated with neonatal sepsis, such as proinflammatory cytokine production, are believed to play a central role in the pathogenesis of this disease. In the present study, serum levels of the proinflammatory cytokines TNF-*α*, IL1-*β*, and IL-6 and the anti-inflammatory cytokines IL-4 and IL-10 were evaluated for 25 subjects with neonatal sepsis. We observed that subjects with late onset of sepsis (LOS), as well as those with early onset of sepsis (EOS), had a substantial increase in serum TNF-*α*. In contrast to EOS, subjects with LOS demonstrated a significant increase in serum levels IL-6 and IL-10. Additionally, we observed a significant difference in cytokine profiles between acute and postacute cases of neonatal sepsis. For instance, the level of proinflammatory cytokines, such as TNF-*α* and IL-6, was elevated in the acute phase, whereas the production of anti-inflammatory cytokines, such as IL-10, became substantially upregulated during the postacute phase. Additionally, no correlation was observed between cytokine levels and CRP levels or lymphocyte counts. Thus, in contrast to CRP levels and lymphocyte counts, examination of the cytokine profile can provide valuable information when determining the most effective therapy for treating neonatal sepsis. This information may be useful to physicians when determining if anti-inflammatory or immune stimulatory therapy is warranted.

## 1. Introduction 

Neonatal sepsis presents a significant health issue and is often associated with a high mortality rate [1]. Very low birth weight infants are especially vulnerable and often tend to develop severe complications, leading to a fatal outcome [2]. Therefore, early diagnosis and implementation of appropriate antibiotic therapy play a crucial role in improving the survival rate of infants with sepsis [3]. The “gold standard” for a diagnosis of the systemic bacterial or fungal infection is the isolation of pathogens from peripheral blood. Unfortunately, the sensitivity of this method is low and thus, a diagnosis of sepsis cannot be excluded even when these results are negative [4, 5].

It is believed that the types of immune responses that occur during a bacterial infection play a central role in the pathogenesis of neonatal sepsis. There are two principal types of such responses: systemic inflammatory response (SIRS) and compensatory anti-inflammatory response (CARS) [6, 7]. Acute-phase proteins such as C-reactive protein (CRP) and procalcitonin (PCT) are known as common biomarkers for SIRS. Accordingly, serum levels of these proteins are significantly upregulated during EOS [8, 9]. Proinflammatory cytokines are also considered sensitive biomarkers of neonatal sepsis. For instance, tumor necrosis factor alpha (TNF-*α*), interleukin-1-beta (IL-1*β*), interleukin-6 (IL-6), and CXCL8 (interleukin-8) levels become rapidly and substantially increased during neonatal sepsis [4, 6, 7, 10, 11]. It is believed that a moderate increase of these cytokines in circulation provides a protective role and promotes an antimicrobial immune response, whereas excessive upregulation of proinflammatory cytokines (often referred to as a “cytokine storm”) is commonly associated with a severe and often fatal outcome due to multiple organ failure [7]. For these reasons, it has been proposed that a serum cytokine profile could be used as a prognostic biomarker to predict the severity of the disease [12].

In the present study, we investigated serum cytokine expression, with respect to disease severity, in subjects with neonatal sepsis. The information afforded by this study may provide useful knowledge for physicians when determining if an anti-inflammatory or immune stimulatory therapy is warranted.

## 2. Methods

*Subjects*. This study was conducted over a period of 10 months between February 2013 and November 2013. In this retrospective study, serum specimens from 25 cases with a diagnosis of neonatal sepsis and eight healthy controls (five full-term newborns and three premature newborns from a period of 32 to 36 weeks' gestation) were provided by the Children's Republican Clinical Hospital of the Ministry of Health, of the Republic of Tatarstan (RHC).

In accordance with the Report of the Expert Meeting on Neonatal and Pediatric Sepsis (8 June 2010, EMA, London) [13], sepsis was defined as the presence of at least two clinical and two laboratory criteria or as a result of suspected or proven infection (positive blood culture). The clinical criteria are (1) body temperature instability; (2) cardiovascular instability; (3) presence of the skin and subcutaneous lesions such as petechial rash or sclerema; (4) apnea or increased oxygen requirement, requirement for ventilation support; (5) feeding intolerance or abdominal distension; and (6) irritability, lethargy, or hypotonia. The laboratory criteria were (1) a white blood cell (WBC) count of <4 or >20 × 10^9^ cells/L; (2) an immature to total neutrophil ratio (I/T) of >0.2; (3) a platelet count of <100 × 10^9^/L; (4) C-reactive protein (CRP) levels of >15 mg/L; (5) blood glucose values of >180 mg/dL or hypoglycemia (<40 mg/dL) confirmed at least 2 times; and (6) metabolic acidosis as characterized by a base excess (BE) of ≤10 mmol/L.

The Institutional Review Board of the RHC approved this study and informed consent was obtained from each subject's respective guardian, according to the guidelines approved under this protocol (Federal Law “Protection of Health Right of Citizens of Russian Federation” N323- FL, 11.21.2011).

### 2.1. Serum

Peripheral blood was collected into serum-separator tubes and separated immediately and aliquots (100 *μ*L) were made and stored at −80°C until being used. Specimens were collected during the first 2 days of the onset of clinical symptoms and laboratory signs of sepsis and seven days later. Control serum samples collected from eight healthy neonates were collected at a single-time point.

### 2.2. Cytokine and CRP Analysis

Serum cytokine levels were analyzed on a Luminex 200 analyzer (Austin, TX) with Millipore Human Milliplex® MAP Single-Plex cytokine kits (Millipore, Billerica, MA, USA). Single-plex kits specific for TNF-*α*, IL1-*β*, IL-4, IL-6, and IL-10 were used in combination according to the manufacturer's instructions. Serum CRP levels were determined using the Randox Full Range CRP immunoturbidimetry assay (Randox Laboratories, Crumlin, Northern Ireland, UK), also according to the manufactures instructions.

### 2.3. Statistical Analysis

Statistical analysis was made using the Kruskall-Wallis and Wilcoxon nonparametric methods with Statistica 6.1 for Windows (Statsoft, Tulsa, OK, USA). Significance was established at a value of *p* < 0.05. Correlation analysis was performed using Spearman method.

## 3. Results 

### 3.1. Study Subject Characteristics

It is well documented that newborns developing EOS become infected during the intrapartum period. For newborns with EOS, 85% of cases present within the first 24 hours, whereas 5% of cases present at 24 to 48 hours, and the balance mostly presents within 48 to 72 hours [14]. Therefore, in our study, we classified the onset of sepsis within the first three days of life as EOS [15]. In contrast, LOS has been defined as infection between 4 and 28 days of life and is due to the horizontal transmission of pathogens during the postnatal period [15].

In the present study, neonates were grouped into two categories: 10 cases presented with EOS and 15 presented with LOS. In both groups, the majority of the neonates were male: 70% with EOS and 60% with LOS. The EOS cohort had five premature neonates (50%), whereas LOS cohort had 10 preterm neonates (66%). Preterm is defined for infants that were born before 37 weeks of gestational and having a birth weight of less than 2.5 kilograms. In the group of newborns with EOS, all preterm infants were born at less than 32 weeks of gestation. In the LOS cohort, 70% of preterm infants were born at less than 32 weeks of gestation, and another three (30%) in the period from 32 to 36 weeks of gestation. Disease patterns of EOS and LOS neonates were as follows: EOS clinically manifested with pneumonia (eight cases), microcirculatory dysfunction (two cases), and urinary tract infection (one case). LOS was associated with pneumonia (seven cases), enterocolitis (six cases), microcirculatory dysfunction (four cases), cholestatic hepatitis (four cases), urinary tract infection (three cases), and pyoderma (two cases) (Table 1). Bacteremia was detected in 12 cases (48%), 10 of which belonged to the LOS cohort. Low percentage of septicemia in the EOS cohort might be due to the early administration of antibacterial therapy.* Staphylococcus epidermidis* and* Staphylococcus haemolyticus* were isolated in EOS cases with septicemia only. In contrast, a plethora of infectious agents were found in LOS cohort and included* Candida krusei* (three cases),* Candida albicans* (two cases),* Staphylococcus epidermidis* (two cases), and one case for each* Klebsiella pneumoniae, Enterococcus faecalis,* and* Staphylococcus haemolyticus* (Table 2). Changes in white blood cell counts (WBCs) included leukocytosis (20% of cases) and leukopenia (40%); 10% of cases have no changes in WBCs. Serum CRP levels were used to examine the inflammatory status. In subjects with sepsis, CRP was higher than the laboratory ranges for healthy donors (1.5 *μ*g/dL). Seven cases (70%) with EOS and 12 cases (80%) with LOS were passively ventilated. Two cases of neonatal sepsis were fatal; each neonate in this category was born with extremely lower body weight. A total of 23 neonates with sepsis successfully recovered.

### 3.2. Cytokine Analysis

**Figure 1:**
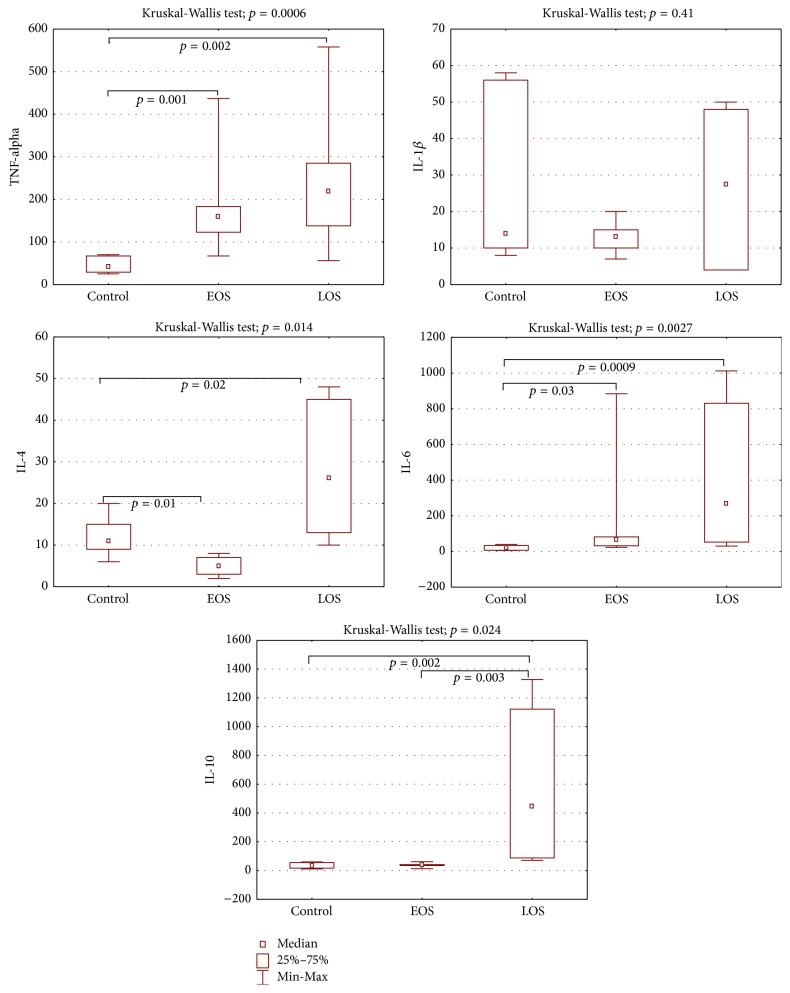
Serum cytokine level (pg/mL) in EOS and LOS types of neonatal sepsis (mean; quartile range). Kruskal-Wallis test was performed for comparison of control group and neonates with EOS and LOS.

Cytokine levels in the control group were not dependent on the gestational age (*p* < 0.05). In contrast to healthy controls, a significant increase in TNF-*α* and IL-6 proinflammatory serum cytokines was observed in LOS and EOS cohorts (Figure 1) (*p* < 0.05). Interestingly, serum level of IL-1*β* did not differ between control and LOS and EOS groups of neonates. In contrast to TNF-*α* and IL-6, level of the anti-inflammatory cytokine IL-10 was significantly different in these two cohorts (*p* = 0.003), as well as between the LOS cohort and healthy controls (*p* = 0.002). Indeed, we observed a significant increase of IL-10 in the LOS cohort only, whereas the levels of IL-10 between controls and EOS cases did not differ from each other. The level of another anti-inflammatory cytokine IL-4 was increased only in LOS cohort (*p* = 0.02). It was found that the fungal sepsis is associated with substantial increase in all cytokines levels (TNF-*α*, IL-1*β*, IL-4, IL-6, and IL-10), when compared with bacterial sepsis (Table 3).

**Figure 2:**
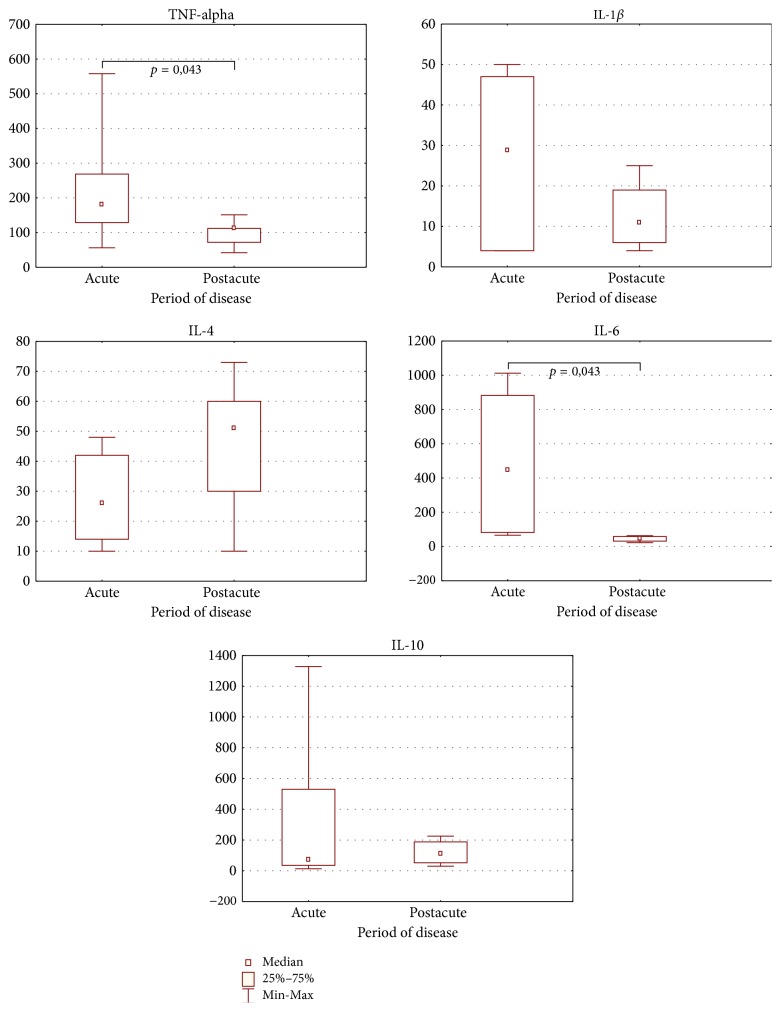
Dynamics of serum cytokine levels (pg/mL) during neonatal sepsis (mean; quartile range). *p* value was calculated by using Wilcoxon method.

To examine the cytokine dynamics of neonatal sepsis, we next stratified cases by those who presented as acute cases and those who presented as postacute cases (Figure 2). For acute cases, the inflammatory cytokines TNF-*α* and IL-6 were significantly upregulated when compared to the postacute cases (*p* = 0.043). Conversely, the anti-inflammatory cytokines IL-10 and IL-4 were upregulated in the postacute phase when compared to the acute cases but it has not been statistically significant (*p* > 0.05). CRP is a pentraxin family protein that is synthesized by the liver in response to factors released by macrophages and adipocytes [14]. Serum levels of CRP rise in response to acute inflammation; consequently, CRP is the most commonly used clinical marker of acute inflammation [16]. To evaluate the association of CRP and inflammatory cytokines in the context of neonatal sepsis, we conducted correlation analysis between CRP and the cytokines TNF-*α*, IL-6, and IL-10 (Table 4). Unexpectedly, we observed no correlation between the CRP and the inflammatory cytokines. We then stratified subjects by the presence or absence of lymphopenia. Cases having a lymphocyte count of <2.0 × 10^9^/L were considered lymphopenic and those with a lymphocyte count of >2.0 × 10^9^/L were considered normal (no cases presented with lymphocytosis). Upon stratification, we observed that levels of TNF-*α* and IL-6 were, on average, increased in the group with lymphopenia and levels of IL-10 were slightly decreased; however, upon correlation analysis we observed no statistically significant correlation between lymphopenia and cytokine levels (Table 5).

## 4. Discussion 

In contrast to previous studies, which reported* Streptococcus group B* as a predominant etiological factor in early neonatal sepsis, we were unable to identify this group of pathogens in our study. The principal reason for this issue might be due to the early initiation of antibacterial therapy (e.g., Ampicillin) in newborns. In our LOS cohort, a low incidence of Gram-negative bacteria in the peripheral blood, as well as* Candida* (33%), might also be related to the early initiation of antibacterial therapy. Moreover, other types of the pathogens present in hospitals and, in particular, intensive care units also might affect the etiology of neonatal sepsis.

Based on the currently accepted viewpoints, the pathogenesis of neonatal sepsis is characterized by a bimodal (i.e., two-phase) immune response [6, 7]. The first phase is predominantly related to SIRS and associated with an excessive release of the proinflammatory cytokines (IL-1*β*, IL-6, IL-8, and TNF-*α*) into the bloodstream. The highest levels of proinflammatory cytokines are referred to as a “cytokine storm” and often associated with a single or multiple organ dysfunctions [7]. The second phase of the immune response is characterized by CARS and is mediated by the secretion of anti-inflammatory cytokines (IL-4, IL-10) [7]. The profound immune suppression in neonatal sepsis is considered to be one of the most important factors of morbidity and mortality of newborns during this period of the disease [17]. TNF-*α*, Il-1*β*, and IL-6 are not considered to be “gold standard” biomarkers of sepsis due to their short half-life [18]. Nonetheless, these cytokines are typically increased very rapidly during neonatal sepsis, even more so the well-known proinflammatory marker C-reactive protein [18, 19]. Previous studies demonstrated procalcitonin, TNF-*α* [10, 20], and IL-6 [21] to be the most sensitive and specific diagnostic markers of neonatal sepsis. We found that the levels of proinflammatory cytokines (IL-6, TNF-*α*) were increased at both EOS and LOS. Importantly, an increase of cytokines levels in our LOS cohort was much more substantial when compared with EOS. This difference might be due to the degree of activation of the immunocompetent cells, known to be frequently involved in pathological processes in the bowel during LOS. The various rates of cytokine increases might be also due to the type of the pathogen, as an etiological factor of sepsis. For instance, it has been shown that fungal sepsis in neonates is associated with substantial increase in IL-6 and TNF-*α* levels, when compared with bacterial sepsis [8, 22]. In our study, fungal sepsis was diagnosed in 33% of LOS cases. Surprisingly, no differences in IL-1*β* levels were found between healthy controls and both groups of infants with NS (EOS and LOS). This fact might be due to the extensive recruitment of the IL-1*β*-producers into affected tissues such as the lungs, bowel, and kidneys. Also, it could be the consequence of the profound dysfunction or suppression of the immune system. Notwithstanding, it is also possible that the mild increase in the proinflammatory cytokines during EOS is due to the immature state of the immune system during the neonatal period [23, 24]. Indeed, the defense mechanisms in neonates are predominantly related to the innate immune reactions, whereas the adaptive immune mechanisms are not yet well established [25]. This fact might be also responsible for the high incidence of NS in premature infants [24]. Intensity of the immune response in neonatal sepsis may be due to decreased expression of innate immunity factors. Reduced expression of the innate immune factors and proinflammatory cytokine synthesis could be due to gene polymorphism that causes a genetic predisposition to various infections, including sepsis [26]. The meta-analysis showed that genetic polymorphisms of IL-1*β* is associated with sepsis susceptibility [27].

It is believed that the expression of anti-inflammatory cytokines usually takes place during the second phase of neonatal sepsis and thus reflects the upregulation of immunosuppressive mechanisms. With this in mind, it is noteworthy that we observed the most significant increase of IL-10 production in the group of LOS neonates. Moreover, we also detected an increase in IL-10 for 20% of cases in the EOS cohort. In an earlier study, IL-10 was reported to be highly sensitive and specific in the diagnosis of neonatal sepsis [28]. These findings indicate that immune reactions associated with EOS and LOS in neonatal sepsis are more complex and do not display a “so-called” bimodal distribution and therefore might develop simultaneously.

Interestingly, the most significant IL-10 increase in peripheral blood was observed for the group of neonatal sepsis subjects with fungal infection, thereby confirming previous findings that fungal infection is commonly associated with immune suppression [29]. Indeed, the frequency of fungal infections in our study was up to 20%. Similarly, an increase of IL-10 in peripheral blood was detected in 28% of cases with neonatal sepsis associated with fungal infections, thus reflecting an immunosuppressive state. The elevated levels of anti-inflammatory cytokines in patients with neonatal sepsis are usually interpreted as a compensatory mechanism, reflecting the activation of systemic inflammation as a response to generalized infection. Accordingly, attempts to suppress innate immune reactions in neonatal sepsis have led to complications such as multiorganic dysfunctions and secondary infections [16]. On the other hand, anti-inflammatory cytokines are known as potent proapoptotic factors [30, 31]. Given that anti-inflammatory cytokines are overproduced in neonatal sepsis, cytokine-induced apoptosis of immune cells might play an important role in immune suppression in neonates with neonatal sepsis [32]. Taken together, these data illustrate the complexity of neonatal sepsis pathogenesis and therefore highlight an importance of the appropriate use of anti-inflammatory and/or immunosuppressive therapy for neonatal sepsis. This is especially true for corticosteroids that are commonly used for neonatal sepsis therapy. On the other hand, the immunosuppression observed during sepsis might be an indication for immune simulative therapy. Therefore, the measurement serum pro- and anti-inflammatory cytokines might be useful in determining a strategy for pathogenic therapy for neonatal sepsis.

## 5. Conclusions 

Sepsis is characterized as a complex and dynamic disease that involves an excessive and suppressed inflammatory and immune response. The immune response in neonatal sepsis associated with proinflammatory and anti-inflammatory cytokines production plays an important role in pathogenesis of this disease. Our data indicates that cytokine profiles provide valuable information for neonatal sepsis therapy and are even more informative when compared with routine CRP and lymphocyte numbers assessment. This information may be useful for physicians when determining if anti-inflammatory or immune stimulatory therapy is indicated.

## Figures and Tables

**Table 1:** Clinical manifestations associated with different types (EOS and LOS) of neonatal sepsis.

Clinical manifestation	Neonatal sepsis	EOS	LOS
	Number of cases (%)	Number of cases (%)	Number of cases (%)
Pneumonia	15 (60)	8 (80)	7 (46)
Pyoderma	4 (16)	2 (20)	2 (13)
Enterocolitis	6 (24)	0 (0)	6 (40)
Cholestatic hepatitis	4 (16)	0 (0)	4 (26)
Urinary tract infection	4 (16)	1 (10)	3 (20)
Microcirculatory dysfunctions	8 (32)	4 (40)	4 (26)

**Table 2:** Pathogenic spectrum of blood-culture proven sepsis episodes.

Pathogen	EOS (*n* = 10)	LOS (*n* = 15)
	Number of cases (%)	Number of cases (%)
*Staphylococcus epidermidis*	1 (10)	2 (13.3)
*Staphylococcus haemolyticus*	1 (10)	1 (6.7)
*Klebsiella pneumoniae*	—	1 (6.7)
*Enterococcus faecalis*	—	1 (6.7)
*Candida albicans*	—	2 (13.3)
*Candida krusei*	—	3 (20.0)

**Table 3:** Serum cytokine level (pg/mL) in confirmed bacterial and fungal neonatal sepsis (mean; quartile range).

Cytokines	Bacterial sepsis	Fungal sepsis	*p*-value
	Mean	Mean	
	(quartile range)	(quartile range)	
TNF-*α*	112.0 (56–177.8)	281.5 (177.5–450)	0.007
IL-1*β*	15.0 (13–27.5)	25.0 (12–39)	0.007
IL-4	43.5 (24.5–46.5)	13.0 (10–26)	0.01
IL-6	26.7 (22.5–41.2)	445.0 (53–491)	0.01
IL-10	88.0 (17–317)	849.0 (323–1225)	0.01

*p* value was calculated by using Wilcoxon method.

**Table 4:** Correlation between serum cytokine (pg/mL) and C-reactive protein (mg/dL) levels.

CRP	TNF-*α*	IL-6	IL-10
	Mean	Mean	Mean
	(quartile range)	(quartile range)	(quartile range)
<3 mg/dL	136.8 (105–342)	219.5 (52–445)	194.0 (35–576)
>3 mg/dL	223.8 (164–285)	477.5 (69–947)	61.0 (41–88)
*R*; *p* value	*R* = 0.15; *p* = 0.57	*R* = 0.38; *p* = 0.26	*R* = 0.18; *p* = 0.6

Correlation analysis was performed using *Spearman* method.

**Table 5:** Relationship between cytokine levels and lymphocyte count.

Lymphocyte count	TNF-*α* (pg/mL)	IL-6 (pg/mL)	IL-10 (pg/mL)
	(Median; quartile range)	(Median; quartile range)	(Median; quartile range)
>2.0 × 10^9^/L	168; 117–231	77; 30–270	102; 35–317
<2.0 × 10^9^/L	253; 149–386	445; 53–882	88; 71–1122
*R*; *p* value	*R* = 0.29; *p* = 0.16	*R* = 0.29; *p* = 0.25	*R* = 0.28; *p* = 0.38

Correlation analysis was performed using *Spearman* method.
